# Supplementary material for: Splice-Junction-Based Mapping of Alternative Isoforms in the Human Proteome
Source: Cell Rep. Author manuscript; Available in PMC 2020 Jan 15. (PMC6961840; doi:10.1016/j.celrep.2019.11.026)
Supplement: 2 [file NIHMS1546469-supplement-2.zip › DF1/PXD010154/Thyroid/Thyroid_21_TG_VMQCLTVAQNNAPSFCPLVVLPSLTEK.pdf]

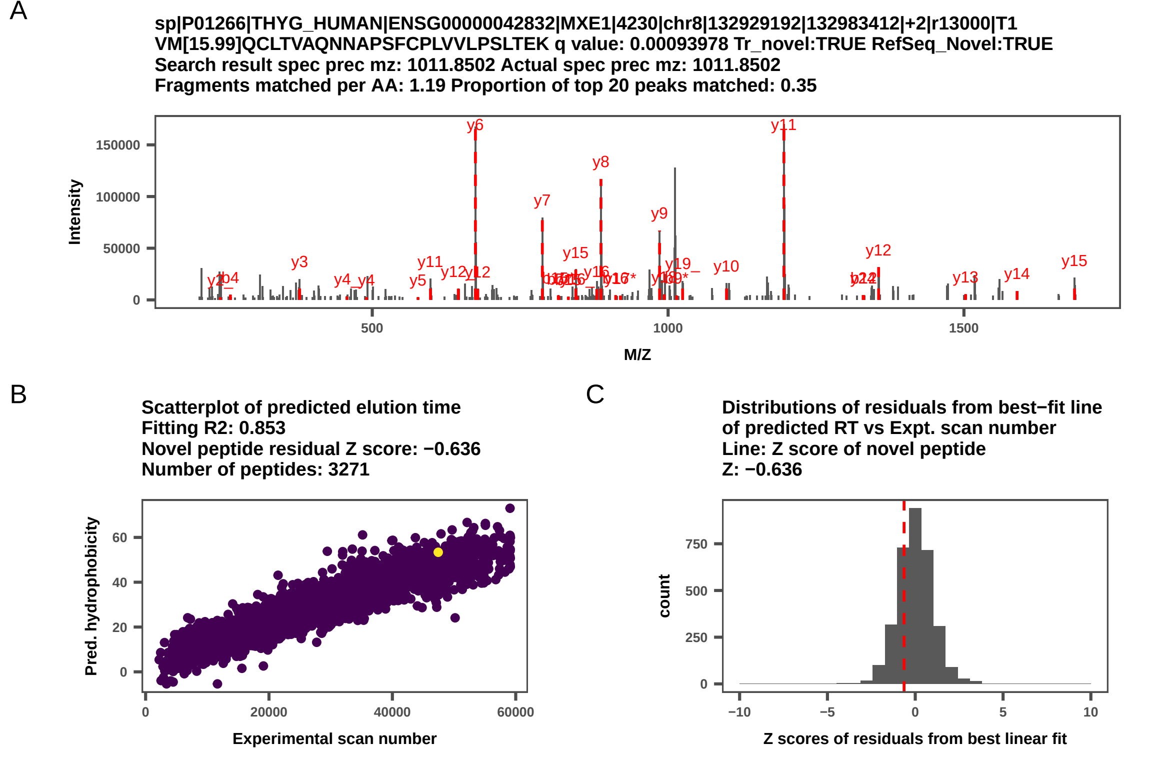
A
sp|P01266|THYG_HUMAN|ENSG00000042832|MXE1|4230|chr8|132929192|132983412|+2|r13000|T1
VM[15.99]QCLTVAQNNAPSFCPLVVLPSLTEK q value: 0.00093978 Tr_novel:TRUE RefSeq_Novel:TRUE
Search result spec prec mz: 1011.8502 Actual spec prec mz: 1011.8502
Fragments matched per AA: 1.19 Proportion of top 20 peaks matched: 0.35
Intensity
150000
100000
50000
0
500
1000
1500
M/Z
y6
y11
y8
y7
y9
y15
y12
y3
y11
y10
y19_
y16
y13
y14
y5
y4_y4
y2_b4
B
Scatterplot of predicted elution time
Fitting R2: 0.853
Novel peptide residual Z score: −0.636
Number of peptides: 3271
Pred. hydrophobicity
60
40
20
0
20000
40000
60000
Experimental scan number
C
Distributions of residuals from best−fit line
of predicted RT vs Expt. scan number
Line: Z score of novel peptide
Z: −0.636
count
750
500
250
0
−10
−5
0
5
10
Z scores of residuals from best linear fit
